# Advances and Limitations of Disease Biogeography Using Ecological Niche Modeling

**DOI:** 10.3389/fmicb.2016.01174

**Published:** 2016-08-05

**Authors:** Luis E. Escobar, Meggan E. Craft

**Affiliations:** ^1^Veterinary Population Medicine, College of Veterinary Medicine, University of Minnesota, St. PaulMN, USA; ^2^Minnesota Aquatic Invasive Species Research Center, University of Minnesota, St. PaulMN, USA

**Keywords:** spatial epidemiology, prediction, fundamental niche, infectious disease, risk map

## Abstract

Mapping disease transmission risk is crucial in public and animal health for evidence based decision-making. Ecology and epidemiology are highly related disciplines that may contribute to improvements in mapping disease, which can be used to answer health related questions. Ecological niche modeling is increasingly used for understanding the biogeography of diseases in plants, animals, and humans. However, epidemiological applications of niche modeling approaches for disease mapping can fail to generate robust study designs, producing incomplete or incorrect inferences. This manuscript is an overview of the history and conceptual bases behind ecological niche modeling, specifically as applied to epidemiology and public health; it does not pretend to be an exhaustive and detailed description of ecological niche modeling literature and methods. Instead, this review includes selected state-of-the-science approaches and tools, providing a short guide to designing studies incorporating information on the type and quality of the input data (i.e., occurrences and environmental variables), identification and justification of the extent of the study area, and encourages users to explore and test diverse algorithms for more informed conclusions. We provide a friendly introduction to the field of disease biogeography presenting an updated guide for researchers looking to use ecological niche modeling for disease mapping. We anticipate that ecological niche modeling will soon be a critical tool for epidemiologists aiming to map disease transmission risk, forecast disease distribution under climate change scenarios, and identify landscape factors triggering outbreaks.

## Introduction

Human history has been shaped by information captured in maps. Concepts such as disease occurrence, epidemics, and outbreaks implicitly have a geographic context. In fact, early stages of epidemiology attempted to understand disease occurrence linking disease cases (e.g., human cholera) with environmental features (e.g., a street pump) in a spatial perspective (Koch and Denike, 2009). Understanding and anticipating the “where” of an outbreak may be a valuable tool for effective public health interventions (Frieden, 2013) as well as for animal health. Thus, disease mapping is key in understanding and anticipating disease occurrence and generating visual tools for decision makers.

Ecology has been proposed as an additional discipline to assist the understanding of why a disease is present in a specific place, but is absent in another (Peterson, 2008). Epidemiology and ecology share goals: the World Health Organization [WHO] (2015) defines epidemiology as “…*the study of the distribution and determinants of health-related states or events*…” while Krebs (1972) defines ecology as the study of the distribution and abundance of species. Terminology for ecology and epidemiology is similar as both disciplines attempt to study and understand the distribution of organisms and their abundance. Such organisms may include plants, animals, or even parasites (i.e., pathogenic or not). At this point, both fields can complement each other; ecology for example has grown through analytical methods and conceptual bases, on the other hand epidemiology has developed an impressive data compilation (Anderson, 1991). Unfortunately, both fields usually work in isolation (Manlove et al., 2016).

Disease biogeography is an emerging field aiming to study the geography of diseases including pathogens, vectors, reservoirs, and susceptible hosts. Disease biogeography links ecology and epidemiology by applying analytical tools from distributional ecology for the study of epidemics. In modern ecology the concept of “niche” is key, thus, we cannot talk about disease biogeography without talking about the ecological niches of a pathogen. This review was inspired by two key publications in disease biogeography: “*Natural nidality of transmissible diseases, with special reference to the landscape epidemiology of zooanthroponoses*” by the Russian Academician Pavlovsky (1966) and “*Biogeography of diseases: A framework for analysis*” by the American Professor Peterson (2008). These pioneers clearly define ideas, terms, and examples of the field of disease biogeography. Pavlovsky and Peterson were the first in developing the use of the ecological niche approach to the study of infectious disease systems, using a diversity of diseases and scenarios to explain why diseases are not distributed at random and that some environmental factors may explain their occurrence in time and space at coarse (i.e., fundamental niche) or local scales (i.e., disease nidus or realized niche). While Pavlovsky’s contribution was observational in nature, Peterson developed a conceptual and methodological framework to develop and interpret quantitative analyses on the biogeography of diseases. Their work can be seen as a theoretical base for applied landscape epidemiology and spatial epidemiology. We aim to provide an overview of current tools and important steps when mapping diseases using ecological niche modeling approaches. This review is intended to serve as an introductory guide for epidemiologists and researchers not familiar with ecological niche modeling techniques.

## Disease Biogeography as a New Paradigm in Epidemiology

Here we will refer to the agents responsible for causing infectious disease as parasites, including micro- and macro-parasites (Hatcher and Dunn, 2011). Some of these parasites may not cause disease in the host (e.g., non-pathogenic strains of *Vibrio cholerae*). In addition to the study of parasites, epidemiologists could be interested also in the vectors and reservoirs (Estrada-Peña et al., 2014) to understand how the parasites are dispersed and maintained in the landscape, respectively. Once ecological features of parasites are defined, their geographic distribution can be expressed in the form of maps, usually in the form of disease risk maps (Peterson, 2008). In this review we will explore the field of ecological niche modeling for understanding disease distribution and posterior disease mapping.

In the late 20th century, in response to the limited unders tanding of disease dynamics from a biological perspective, a new paradigm was proposed in epidemiology: eco-epidemiology (Susser and Susser, 1996a). Eco-epidemiology is based on the need to understand infectious diseases using an ecological approach to help anticipate disease distribution. Although almost three decades have passed since this idea was formally proposed, there are still ambiguities in this approach. While Susser and Susser (1996b) correctly state that disease systems include a set of interconnected environmental factors, they did not define and delimit the eco-epidemiology concept *per se*, which could be a cause of the slow adoption of this term more broadly in epidemiology and ecology (only ∼200 articles in 20 years with this term were found in PubMed). The initial description of eco-epidemiology includes the *Chinese boxes* idea, stating that disease systems are a set of factors with a coherent hierarchy structure, thus, alteration in the parasite system may cause disease only if factors at higher levels of the structure are affected (Susser and Susser, 1996b). However, from a biological perspective, this approach seems inaccurate considering that in nature, when considering parasites in ecosystems, there is not a chain-like configuration. Instead, parasites in natural communities interact with several species in the food web, thus, parasite systems appear as part of an interconnected network of species (Hudson et al., 2006). This was exemplified by Pavlovsky (1966) who used a series of examples on the study of infectious diseases including yellow fever (*Flavivirus*), plague (*Yersinia pestis*), tularemia (*Francisella tularensis*), and leishmaniasis (*Leishmania* spp.) to demonstrate the complexities in the biological interactions of parasite systems. Later, Peterson (2008) proposed disease biogeography as the branch of biology related to the geography of infectious diseases; disease biography aims to identify the factors associated with disease occurrence allowing us to understand and potentially predict epidemics.

## The Ecological Niche of Parasites

### The Term Niche

Infectious diseases are, by definition, the complex association between at least two organisms: pathogen and host. Infectious disease requires the presence of all key actors in a disease transmission system (e.g., parasite, vector, susceptible host; Peterson, 2006b). Identifying the environmental factors which allow the presence of one of these actors in the disease system elucidates the ecology and geography of a specific infectious disease (Peterson, 2007). Recognizing patterns of species distributions and identifying the specific environmental requirements of species to persist in the long term has been extensively studied in ecology with an empirical and theoretical body that supports the study of distributional ecology of organisms. However, disease biogeography is just one component of multifaceted questions in understanding the ecology of parasite transmission, and has been poorly addressed in biodiversity research (Peterson et al., 2011).

A disease system may include a pathogen species, a vector species, and a host, or it may be more complex including a vast number of competent vectors and host species in the same locality, sharing the environmental conditions suitable for them, sharing their *ecological niches* (Soberón, 2007). Furthermore, the parasites’ ecological niche is linked to its geographic distribution (Soberón and Nakamura, 2009). Establishing environmental variables able to track areas of potential distribution of diseases was proposed in epidemiology in the mid-20th century, using rudimentary techniques to correlate parasites with environmental factors. It was found that diseases do not occur randomly in space; hence the concept of infectious disease *nidality* was defined in epidemiology as the feature of an infectious disease to be constrained under specific environmental conditions (Pavlovsky, 1966). The words *niche* and *nidality* have the root word *nidus* which means nest. The association between the parasite’s suitable environments and geographic ranges is the base of the ecological niche modeling field. If the environmental factors suitable for a parasite are available outside the known range, an epidemic may appear in a novel (suitable) area. This phenomenon is well known in invasion biology (Peterson and Vieglais, 2001), while in epidemiology the invasion by parasites is known as *pathogen pollution* (Anderson et al., 2004) but has not been explored quantitatively in detail.

**FIGURE 1:**
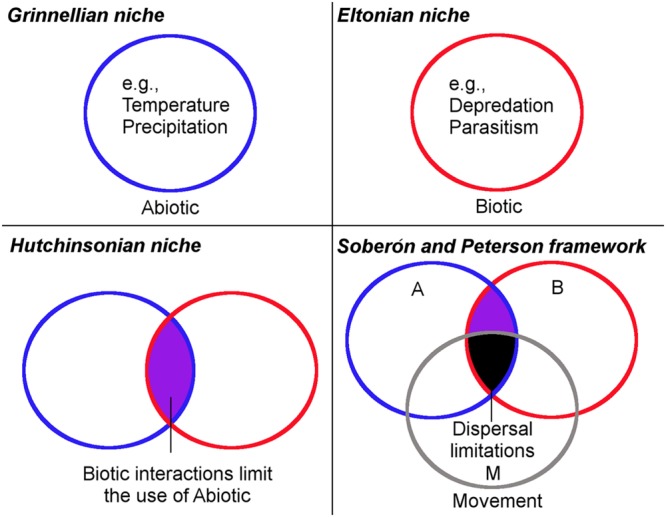
**Historical framework of the ecological niche concept. (*Grinnellian niche*)** The ecological niche idea originally focused on the abiotic factors delimiting an organism’s occurrence. Under this scenario, all the suitable abiotic conditions in the circle are accessible for the parasite. **(*Eltonian niche*)** Ecological niche idea considered the parasite’s role in the ecosystem and its interaction with other organisms. Under this scenario, the entire area inside the circle is suitable and accessible for the parasite. **(*Hutchinsonian niche*)** Ecological niche idea considered the abiotic factors limiting the parasite’s presence (fundamental niche) and the biotic interactions limiting the parasite’s presence (realized niche). Under this scenario, all the overlapping area between circles (purple) is abiotically and biotically suitable and accessible for the parasite. **(*Soberón and Peterson framework*)** The modern ecological niche framework considering the access of the parasite to abiotic and biotic factors allowing it to survive. Under this scenario, the overlapping area between circles is abiotically and biotically suitable for the organism, but due to dispersal limitations it occupies only a portion of potential suitable areas (black). Blue denotes the abiotic factors **(A)**, red represents the biotic factors **(B)**, while gray denotes the movement and dispersal capacity **(M)** of the organisms to use the suitable areas. Modified from Peterson et al. (2011).

The term “niche” was used in ecology by Grinnell (1917) to refer to the combination of environmental factors present in a species’ range; he discussed how these factors may restrict the distribution of species. Even though the word niche was first used for a bird, the concept was successfully adopted by ecologists and is today a key concept in ecology. However, the niche concept suffered from ambiguity and incorrect use (Godsoe, 2010; McInerny and Etienne, 2012; Warren, 2012). In fact, the concept had four main stages before its current definition (**Figure 1**). Grinnell (1917) used the term niche to refer to the environmental factors required by a species for its distribution (*Grinnellian niche*; **Figure 1**). Then years later Charles S. Elton defined niche as the role of a species in an ecosystem and its interactions with other species (*Eltonian niche*; **Figure 1**). Grinellian and Eltonian definitions clearly were based on different points of view. G. Evelyn Hutchinson attempted to reduce the ambiguity of the niche concept, differentiating it as the fundamental niche and realized niche (*Hutchinsonian niche*; **Figure 1**). The fundamental niche was proposed as a hypervolume of environmental variables that allow the species to exist without immigration, while the realized niche incorporates the idea of the portion of the fundamental niche actually used by the species due to negative (e.g., competition) or positive (e.g., facilitation) biological interactions with other organisms (Bruno et al., 2003). Finally, Soberón and Peterson (2005) defined the niche concept using the **BAM** framework and a body of empirical and theoretical background explaining this framework. Thus, the current ecological niche term refers to the environmental conditions in which a species can maintain populations in the long term without need of immigration. The species, however, may not use its entire niche due to biological or dispersal limitations. According to Soberón and Peterson (2005), a missing component in previous definitions of niche was the dispersal capability and movement potential of species to reach suitable areas (BAM framework of *Soberón and Peterson*; **Figure 1**). They suggest that a species may have a broad fundamental niche, but may be unable to use it entirely, due to biogeographic limitations (e.g., mountains, rivers, oceans acting as barriers). All the different niche concepts reflect the considerable debate to define the term; now a clear and delimited definition of a niche is available and employed in ecological niche modeling (Warren, 2012).

### The “BAM” Framework in Disease Systems

The **BAM** diagram incorporates the dispersal capacity of parasites when describing their niches. Dispersal abilities of parasites are key to understanding disease distributions. For example, chikungunya virus (*Alphavirus*) was absent in the Americas prior to 2013 due to an ocean functioning as natural barrier between Asia and the Americas (Van Bortel et al., 2014). However, global movement of humans allowed dispersal of chikungunya into South, Central, and North America, as well as the Caribbean, and resulted in a successful virus invasion. This invasion demonstrates that the Americas have environmental conditions falling inside the ecological niche of chikungunya, but the virus was not previously present in the Americas due to dispersal limitations. Once dispersal limitations are overcome or the natural population dynamics change, pandemics may appear (Pavlovsky, 1966; Hatcher and Dunn, 2011). To understand which areas have the potential for disease dispersal under an ecological niche approach, the **BAM** diagram is a powerful tool that helps to (i) understand ecological processes, (ii) design studies, and (iii) interpret experimental, virtual, or field research (Soberón and Peterson, 2005; Peterson, 2008). Peterson et al. (2011) discuss in detail the modern use of the ecological niche concept using examples and mathematical terms. Here we briefly describe the **BAM** framework.

#### “B”

**B** refers to biotic factors shaping the distribution of the parasite (i.e., biocenose *sensu* Pavlovsky, 1966, or binomic *sensu* Hutchinson; Soberón, 2007). This is a critical component in the ecological niche of parasites considering that biotic interactions between hosts and vectors may promote or limit parasite occurrence even in environmentally suitable (i.e., **A**) and accessible areas (i.e., **M**). Some biotic factors such as host nutrition, density, behavior (e.g., cultural practice of kissing dead bodies that may promote Ebola infections; Woźniak-Kosek et al., 2015), and co-infections may benefit parasite presence. On the other hand, some biotic factors may limit parasite occurrence including host immunity (e.g., acquired immunity to *V. cholerae* after an epidemic will reduce cases in the next cholera outbreak; Koelle et al., 2005) and behavior (e.g., use of protective measures to avoid sexually transmitted diseases). The biotic component is critical to understand the ecology of parasite transmission, and their effects are evident when developing studies at fine geographic scales.

Pavlovsky (1966) studied the occurrence of parasites using an ecological niche approach and proposes the concept of “*micronidus.*” The micronidus is the term for the biotic factors indispensable for the parasite’s cycle at a very fine scale; such factors, however, should not affect estimations of the ecological niche at coarse scales (Pavlovsky, 1966). The micronidus simply occupies a specific portion of the parasite’s niche. Peterson et al. (2011) identify these factors acting at a local scale and also ignore them when modeling a species’ ecological niche at a coarse scale with successful predictions. Empirical evidence suggests that the micronidus may not be key for the parasites’ ecological niche in general terms (Maher et al., 2010), such as when considering climate conditions. Assuming that local biological interactions are meaningless at coarse scales is referred to as the “*Eltonian Noise Hypothesis*” (Peterson et al., 2011). The idea behind this hypothesis is that biotic interactions at the individual level (e.g., host immunity) or the microhabitat required by a specific phase in the parasite cycle (e.g., the humidity in the host burrow required during the metamorphosis of *Phlebotomus* vectors) may play a minor role when estimating the parasites’ niche at coarse scales. Furthermore, biotic interactions are important only when studying diseases at very fine spatial scales such as when studying transmission dynamics within a population (Peterson et al., 2011). This series of evidence and assumptions supports the idea of mapping diseases based on climatic variables or other environmental features.

In fact, when estimating the ecological niche of a generalist parasite, it is evident that climate conditions are crucial for parasite establishment and biotic interactions may be ignored at coarse scales. For instance, plague bacterium occurs in consistent and measurable climatic conditions; in other words, the environmental signature allows us to predict plague occurrence in North American wild mammals with no information about biological interactions (Maher et al., 2010). For example, Maher et al. (2010) suggest that, after assessing 72 plague reservoirs, plague occurs under predictable spatial and environmental situations, and host species involved in the transmission cycle are less relevant to maintain the parasite permanence than climate. Parasites with broad niches (i.e., generalist species) may maintain disease cycles under diverse environmental conditions and consequently may affect a broad range of taxa (e.g., plague, influenza (*Influenzavirus A*), leptospira (*Leptospira* spp.); Pavlovsky, 1966; Tong et al., 2012). Parasites that use different species of hosts and vectors are termed *polyhostal* and *polyvectored* respectively (Pavlovsky, 1966) and can be modeled including all the actors in the system or based on disease cases only (Peterson, 2007).

Biological interactions between species at very fine scales are complex. In co-infections, two parasite species within a host may even interact; one parasite may limit the presence of other parasite species (Pavlovsky, 1966). For example, after *in vivo* experiments of multiple inoculations of the parasites *Brucella suis* and *Coxiella burnetii* in guinea pigs, Mika et al. (1959) suggested that one parasite species may show apparent competition-like interactions. In such experiments, infected guinea pigs showed faster recovery or even unapparent infections of *C. burnetii* when the *B. suis* was present, as opposed to those with single infections —suggesting that the presence of *B. suis* is protective for *C. burnetii*. This mechanism is recognized and used in the poultry industry through the use of non-pathogenic bacteria to promote competitive exclusion against pathogenic strains of *Salmonella* spp. (Revolledo et al., 2006).

#### “A”

The **A** factor on the **BAM** diagram represents the abiotic conditions limiting survival of parasite populations in the long term (i.e., geobiocenose *sensu* Pavlovsky, 1966, or scenopoetic *sensu* Hutchinson; Soberón, 2007). The area of overlap between the abiotic factors **A** with biotic factors **B** denotes which factors allow for parasite presence (**Figure 1**). Examples of abiotic conditions critical for parasite survival may include temperature and humidity (e.g., bacterial diseases in plants), solar radiation (e.g., viruses outside the host), and soil chemistry (e.g., fungi). Thus, abiotic environmental factors allowing for the occurrence of parasites in a specific location can be measured at diverse spatial scales.

#### “M”

Parasite occurrence may be constrained due to limited dispersal abilities (e.g., short movements of soft ticks) and biogeographic barriers (e.g., oceans). This key concept is termed movement or **M** in the **BAM** framework and considers the geographic accessibility of organisms. Changes in **M** affect the parasite’s distribution and can be expressed as limitations of accessibility (e.g., dengue virus was absent from Pascua Island due to the isolation of this island, despite the vector being abundant; Perret et al., 2003) or an increased potential of accessibility (e.g., ballast waters increased dispersal of *V. cholerae*; McCarthy and Khambaty, 1994).

Identifying the environments suitable for an organism (**A**) is feasible at different spatial scales from petri dishes to continental extents (e.g., *V. cholerae*; see Huq et al., 1984 and Lobitz et al., 2000). Characterization of the biotic component (**B**), allowing for a parasite presence or absence, is much more complicated due to temporal-spatial dynamics and complexities of biotic interactions. In ecological niche models of large taxa (e.g., birds) the **B** component is usually neglected, ignoring biotic interactions in view of the robustness in predicting species occurrence, and based on the assumption that biological interactions are indistinguishable at coarse scales (Peterson et al., 2011). For parasites, however, their strong dependence on other organisms (i.e., the host) makes considering biotic interactions important in estimating their areas of occurrence. Thus, the more we understand about the natural history of a disease, the better the ecological niche estimation and interpretation of model outputs; however, modeling such complexities could be a challenge.

Parasites occur naturally with animals and plants and have key roles in ecosystems. The presence of a parasite in a host does not necessarily represent disease. In fact, there is growing evidence that parasite diversity is an indicator of ecosystem health (Hudson et al., 2006). When anthropogenic perturbations alter parasite cycles or communities, disease outbreaks can appear (Hatcher and Dunn, 2011). Parasites are usually considered negative in the context of human populations. In fact, a considerable amount of literature related to the distribution of parasites is developed only under disturbed/epidemic events; therefore limited knowledge exists about parasites in natural and non-disturbed conditions. Human societies should maintain pristine areas as reserves, including the greatest variety of biomes possible, to understand parasite ecology for epidemic prevention purposes (Pavlovsky, 1966).

## From Disease Reports to Disease Maps

The environmental space that a parasite is occupying (i.e., the existent fundamental niche; Peterson et al., 2011) can be expressed in terms of geography. By identifying the suitable environmental conditions for a parasite, we can identify the areas in which a parasite can maintain populations in the long-term; this helps to understand the geographic distribution of parasites (Peterson, 2006b). Due to this link between the niche and the distribution of species, the terms ecological niche modeling and species distribution modeling are often used as interchangeable terms. However, niches are characterized in an environmental dimension, while geographic distributions are the expression of the ecological niche in the geography (Warren, 2012).

### Current Methods for Disease Mapping

Techniques to show disease distribution include choropleth maps (i.e., coloration of political/administrative units with colors according to categories of incidence, prevalence, or risk rates established *a priori*) and proportional symbols (i.e., symbols like circles with sizes classified according to predefined disease occurrence categories). These methods are data descriptive and easy to read and interpret, but fail to anticipate the parasite occurrence in areas where no data are available.

Analytical tools have improved since early disease mapping in the 19th century (Carpenter, 2011). However, when comparing John Snow’s historical 1854 cholera map with current disease mapping based on density analyses with novel tools and software (e.g., Le Comber et al., 2011), it is evident that mapping approaches in epidemiology have not substantially improved. Indeed, epidemiology is dominated by studies using spatial density of cases, spatial interpolations of reports, and geographic distances to identify areas of potential disease-transmission risk (Auchincloss et al., 2012). These epidemiological techniques are a powerful source of information to show patterns of disease surveillance and reporting effort; however, they have several limitations in predicting disease risk. In fact, because spatial interpolation maps base estimations on available geographic coordinates solely, they should be considered as surveillance-effort maps instead of disease-transmission risk maps.

Using maps based solely on spatial interpolation to identify disease risk could be challenging. Mapping methods using spatial distances usually base their analyses on straight lines of geographic or even Euclidean distances (Auchincloss et al., 2012), which may fail to capture the biological realism of disease systems. Thus, spatial interpolation and cluster analyses are in essence data driven and prone to sampling bias effects. Spatial interpolations may attribute low parasite occurrence to an area with no data. This type of analysis is thus prone to miss areas of high disease risk because of surveillance gaps. For example, poor countries with limited epidemiological surveillance may appear healthier due to zero (i.e., lack of) cases reported, however, the real situation may include high disease incidence.

**FIGURE 2:**
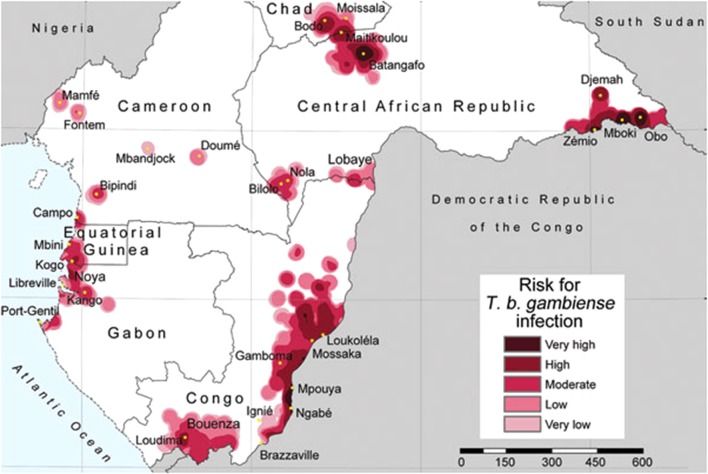
**Trypanosomiais in central Africa based on density of reports**. Areas with more reports are defined by the authors as of very high risk, while areas with limited or no data are defined as very low risk of infection. Note that the estimation of “risk” was truncated based on administrative boundaries resulting in lack of information for the Democratic Republic of the Congo. Figure from Simarro et al. (2011) published under the Creative Commons Attribution (CC BY) license.

For example, recent research proposed risk levels of human Trypanosomiasis, a vector-borne protozoan parasite (Simarro et al., 2011), where risk estimations were based on the spatial density of human cases reported. To define close and distant cases authors proposed a 30 km radius—a pragmatic value neglecting the biology of the vector. In the Trypanosomiasis study, areas with high number of reports are defined as of “very-high risk,” while areas with no data are simply ignored by the model and assumed in the “very-low” or no risk categories (**Figure 2**). Additionally, the risk estimation was restricted to administrative boundaries, failing to include the natural history of this disease caused by tsetse flies of the *Glossina* genus. This error was later replicated with the same data and method by Simarro et al. (2012) adding new countries. Both publications resulted in two isolated studies that did not provide a complete history on the biogeography of this disease in central Africa, but more importantly the studies proposed no risk in broad areas where no information is available, which may result in an incomplete or incorrect message to public health authorities.

Spatial interpolations for disease mapping, including kernel smoothing and kriging, attempt to describe biological mechanisms driving parasite spread among populations, but are strongly biased by surveillance effort (e.g., if most data were collected close to roads; Kadmon et al., 2004). Because disease maps may be used to guide surveillance and disease management (Stevens and Pfeiffer, 2011), controlling, or at least recognizing, sampling bias is critical. Additionally, spatial interpolations based solely on geographic coordinates assume that the landscapes where parasites occur are environmentally homogeneous, failing to provide explanations for the environmental processes and landscape variables triggering or limiting outbreaks.

### Spatial Interpolation vs. Environmental Interpolation

To improve on the limitations of disease maps based on density and distance of geographic coordinates solely, ecologists started linking environmental variables with disease occurrence. Thus, environmental interpolations can be an alternative to spatial interpolations (Peterson, 2014). Environmental interpolations are the core approach in ecological niche modeling and include two main characteristics. The first is descriptive; ecological niche models attempt to identify the environment associated with the parasite’s occurrence in the field or via laboratory experiments of physiological tolerance to specific environmental variables (Peterson et al., 2011). The second characteristic is predictive, searching across areas of interest to identify environmental combinations similar to those where the parasite occurs. Thus, while geographic interpolations occur in the geographic space, environmental interpolation is developed in multidimensional environmental scenarios. By using ecological niche modeling techniques we gain knowledge on the association of organisms with environmental variables of interest, contributing to our understanding of the parasite’s ecology and geographic distribution (Peterson, 2006b). Additionally, with the knowledge obtained from few observations, inferences may allow us to identify areas environmentally suitable for the parasite in areas without reports available (Peterson et al., 2004).

**FIGURE 3:**
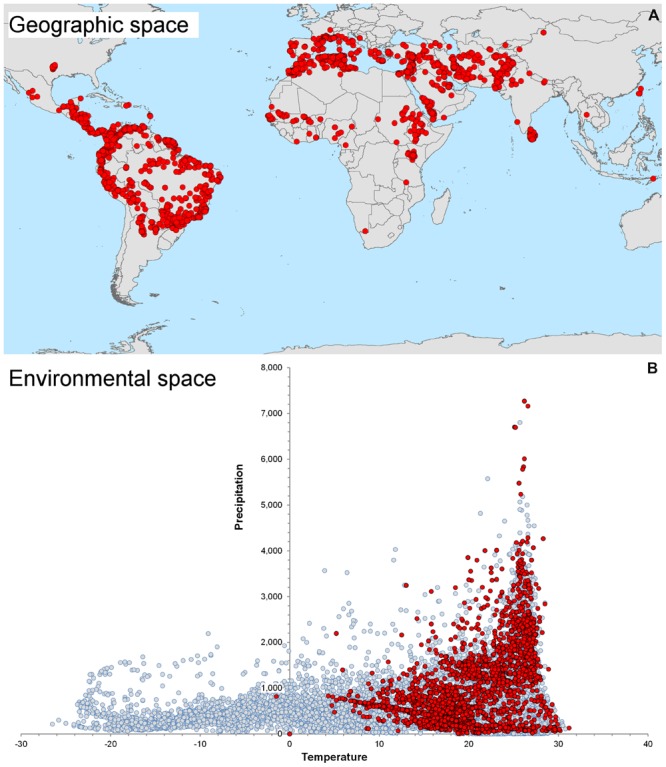
**Global distribution of cutaneous leishmaniasis. (A)** Visualization of 6,426 cutaneous leishmaniasis occurrences (red points) in the geographic space. **(B)** Distribution of leishmaniasis in the environmental space. Some occurrences have identical environmental values and therefore resulted in 1,954 single occurrences in the environmental space (red points). Notice the diversity of environments available across the globe (gray points) and the consistent, narrow, predictable environmental space occupied by the disease. The environmental space (gray points) was generated using 10,000 random points globally to capture values of temperature (*x* axis) and precipitation (*y* axis). Data obtained from Hijmans et al. (2005) and Pigott et al. (2014a).

**FIGURE 4:**
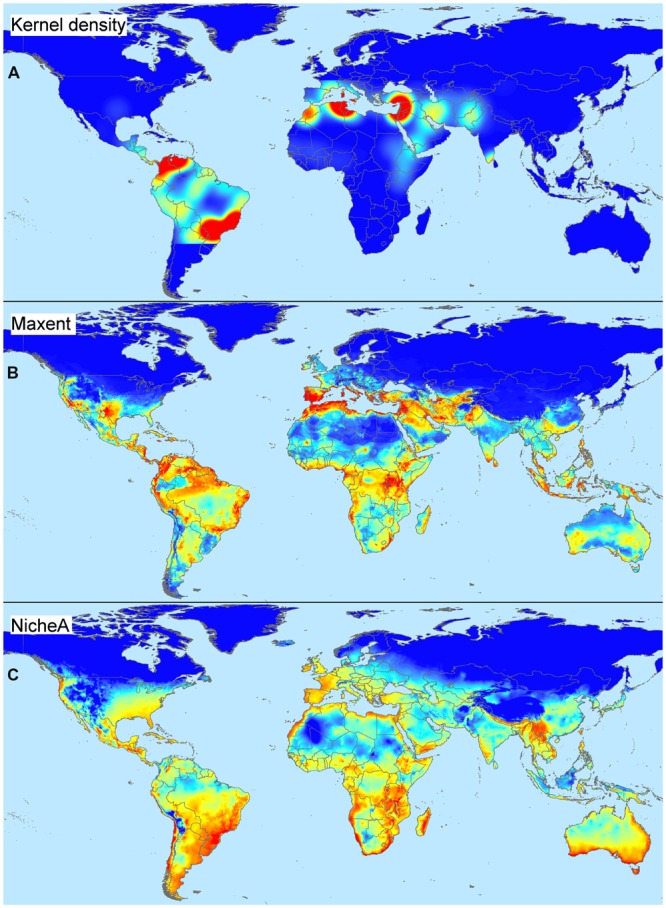
**Spatial and environmental interpolations of cutaneous leishmaniasis. (A)** Kernel density estimation based on leishmaniasis occurrences from **Figure 3A** falling in a pre-determined occurrence-distance radius, no environmental conditions are considered. Model constructed using default parameters in ArcGIS 10.2 (ESRI, 2015). Notice that areas proposed as of risk (red) are overfitted to locations with abundant disease reports, while areas with low or no reports are denoted as of low importance (see similarity with map in **Figure 3A**). The model does a poor job of predicting areas where data are absent (e.g., truncated areas in South America). **(B)** Environmental suitability index based on environmental similarity with sites where leshimaniasis was reported; environmental conditions are considered in this model. Model constructed using default in Maxent 3.3.3.k (Phillips et al., 2006) and bioclimatic variables Bio1 – Bio7 and Bio10 – Bio17 cell size of ∼4.5 km (Hijmans et al., 2005). Notice that suitable areas for disease occurrence are predicted in areas with a lack of data. Areas of high suitability, however, mirror the areas with abundant disease reports; this is a form of model overfitting based on environmental values. **(C)** Environmental suitability index based on distance to the niche centroid; environmental conditions are considered. Model constructed using default parameters in NicheA 3.0 with the same environmental variables as above (Qiao et al., 2015a). Notice that suitable areas are estimated in the environmental space thereby reducing spatial overfitting; predictions do not reflect the number of occurrences, but their position in an environmental cloud provides the highest values to points in central areas of the environmental space and low values to most external disease reports (see distribution in the environmental space in **Figure 3B**). Estimations of low (blue) and high (red) represent values ranging between 0 – 7.25 (Kernel density), 0 – 0.86 (Maxent), and –1 – 0.99 (NicheA). Data obtained from Hijmans et al. (2005) and Pigott et al. (2014a).

We highlight the differences between spatial and environmental interpolation using data from the global burden of cutaneous leishmaniasis (Pigott et al., 2014a). The 6,426 cutaneous leishmaniasis occurrences were plotted in the geographic space using latitude and longitude as coordinates and then were plotted in the environmental space using temperature and precipitation as coordinates (**Figure 3**). This procedure reduced the original 6,426 geographic coordinates to 1,964 single coordinates in environmental dimensions, allowing identification of the environmental space used by the species. We then modeled the potential areas for the occurrence of this vector-borne disease using spatial and environmental interpolations (**Figure 4**). First we developed maps based on simple geographic interpolation using a density kernel estimation that identifies areas with high or low number of occurrences under a specified radius. Then we modeled the ecological niche of the disease using two different methods: Maxent, that identifies the association between occurrences and environmental variables weighted by the number of occurrences, and NicheA, that identifies the environmental space occupied by occurrences and weights the occurrences based on their position in the environmental space, thereby mitigating the effect of oversampled areas (**Figure 4**). In this exploration, spatial interpolations were restricted to denote high values only in areas with adequate data. The ecological niche models from Maxent and NicheA, based on environmental interpolations, found areas suitable for potential leishmaniasis occurrence even in areas with gaps of surveillance.

Ecological niche modeling is now commonly practiced in ecology and there are a number of sophisticated niche modeling tools available to analyze a wide range of datasets. Modeling techniques that link parasite occurrence with environmental variables include: (i) those requiring presence-only data like Bioclimatic Envelop Algorithm (BIOCLIM), Ecological Niche Factor Analysis (ENFA), and Niche Analyst (NicheA); (ii) regression models requiring presence plus true absences or pseudoabsence data as Boosting Regression Trees (BRT), Classification and Regression Trees (CART), Generalized Linear Models (GLM), Generalized Additive Models (GAM), and Random Forest (RF); and (iii) algorithms requiring presence-background data including Maximum Entropy (Maxent), *n*-dimensional hypervolume (Blonder et al., 2014), and Genetic Algorithm for Rule-set Production (GARP). There is no “best algorithm” that fits with all study case configurations. Instead, several algorithms must be assessed in each study case to identify those performing well under the specific conditions of the disease system and available data (Qiao et al., 2015b). These modeling algorithms have been explained and discussed with more detail elsewhere (Elith et al., 2006; Franklin, 2009; Peterson et al., 2011).

## Reports of Disease Presence

**FIGURE 5:**
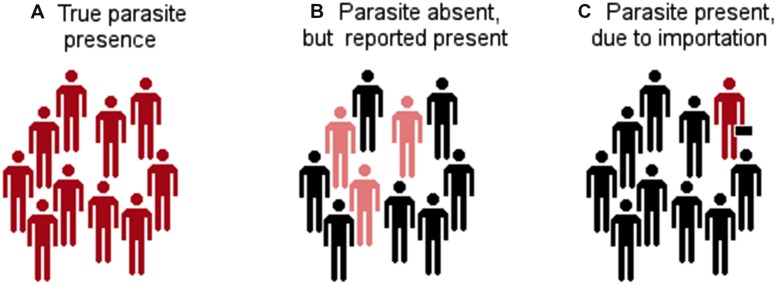
**Types of parasite presence reports. (A)** The parasite was identified in a host population and it is reported as present. **(B)** There are incorrect reports of parasite occurrence due to error in the identification of other parasites that generate cross reactivity to the diagnostic test. **(C)** Reports of parasite occurrence but the infections occurred in a different location to that included in the site of diagnosis (e.g., from a traveler). Red: infected with the parasite. Pink: not infected with the parasite but seropositive. Black: not infected.

Ecological niche modeling generally needs records of sites where the parasite is present to link the parasite’s occurrence with the environmental features chosen by the researcher. Presence records are critical and need to be accurate in terms of parasite identification and geolocation. However, reports of parasite presence may include some level of uncertainty (**Figure 5**). For example, the parasite may be present in a site and it could be correctly identified and georeferenced. But in some instances the parasite may be reported as present in a location when in reality the parasite is absent. This could be due to incorrect diagnostic tests. This occurs with parasites that have sympatric and taxonomically close species with similar morphological or immunological characteristics. Another confounding factor is the report of the presence of a parasite in a site where, in fact, suitable conditions do not exist. This may occur in situations where the parasite was translocated by the host. For example, a human infected with Ebola in Africa can move to the Arctic in less than 24 h, in this simple case, reporting the disease detection in the Arctic may generate estimations that do not resemble the parasites’ niche. Thus, using reports of parasite’s presence including errors of identification and site of infection will generate inaccurate risk models. Additionally, georeferencing accuracy is an issue of disease mapping that deserves critical attention (Auchincloss et al., 2012), but has been neglected when modeling the potential distribution of infectious disease (but see Nakazawa et al., 2010; Peterson and Samy, 2016).

**FIGURE 6:**
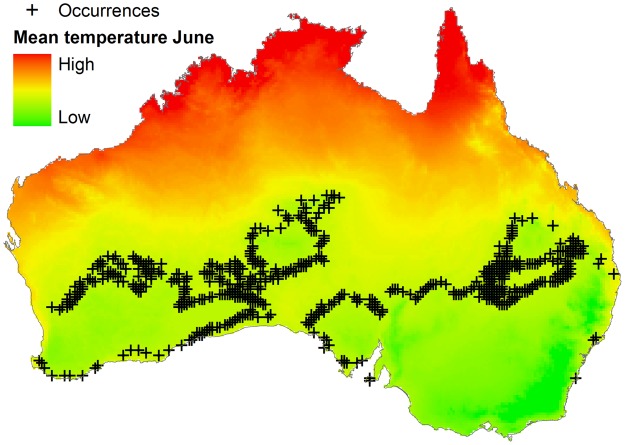
**Distribution of occurrences based on a geographic dimension**. Occurrences of a virtual parasite (*n* = 636) are dispersed across Australia (black crosses). Mean temperature for June is variables in the country (background colors), occurrences however, do not have environmental independence: all points fall in sites with identical temperature values (i.e., 12.5°C). Temperature data obtained from http://www.worldclim.org/ (Hijmans et al., 2005).

Algorithms could be calibrated using parasite, vector, or reservoir occurrences plus environmental information. Data on reservoir occurrence may come from the researcher’s fieldwork, scientific literature, natural history museum collections, official public health agencies, and laboratories. Data for vectors exist but are scarce compared to data for vertebrate reservoirs (Peterson, 2014). Data for parasites are scarce and can be generated by the researcher or can be obtained from health agencies, scientific literature, or online repositories like Healthmap^1^, but need considerable data cleaning to reduce errors and uncertainty (Peterson, 2014). Georeferencing error in occurrence points and distance between them is also informative when determining the environmental variables required for ecological niche modeling. No magic recipe exists to establish the ideal or minimum number of reports for ecological niche model calibration; it simply depends on the research question, study design, the environmental variables considered, and data available. Something to keep in mind is to avoid the modeler’s spatial-bias (i.e., bias implicit when thinking in the geographic space neglecting environmental dimensions; see **Figure 3**). For example, the number of occurrences used for model calibration could be numerous in the geographic space, but may be meaningless when considered in the environmental space. To show this, more than 600 occurrence points were generated for a virtual parasite in mainland Australia, but when such points are considered in environmental terms, for example the mean temperature in June, all points have the same mean temperature value (i.e., 12.5°C; **Figure 6**). Thus, these abundant points from a geographic perspective represent a single point in environmental terms.

**FIGURE 7:**
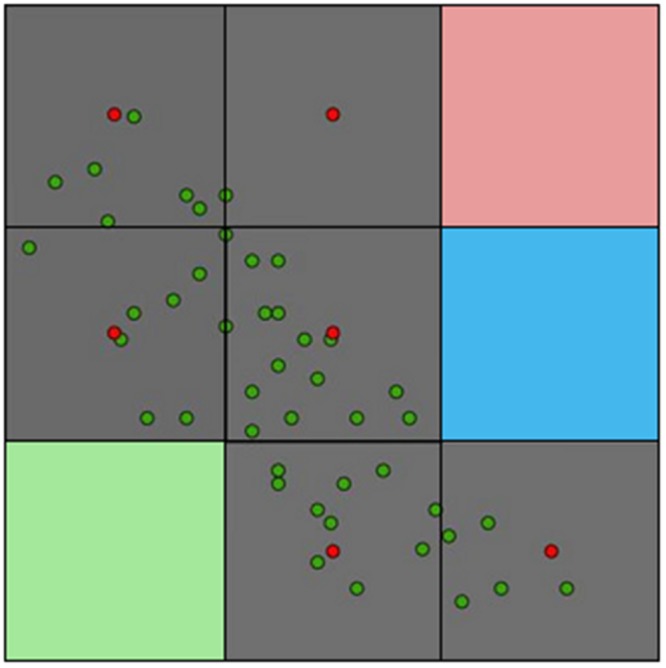
**Example of occurrences represented in geographic and environmental terms**. Original reports of disease (green points) can be an overrepresentation of environmental conditions associated with sampling bias. The study area (grid) may require a resampling strategy to obtain only one report per environmental cell to mitigate model overfitting in oversampled areas (red points). In this study area four environmental values are present: gray, pink, blue, and green. A more strict modeling approach (e.g., Qiao et al., 2016) would require only one point per environmental value. Thus, in this example, only one value representing the occupied environment (i.e., gray) should be considered for modeling purposes. Source (Escobar and Peterson, 2013).

**FIGURE 8:**
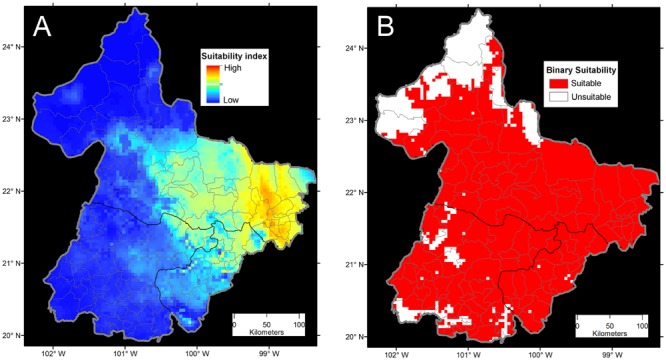
**Ecological niche model comparisons of bat-borne rabies in cattle in Mexico. (A)** The continuous model reflects the sampling effort in terms of numbers of rabies cases available (not shown) and high model overfitting from using all the 19 bioclimatic variables from Worldclim (Adapted from Bárcenas-Reyes et al., 2015). Risk was defined in terms of Maxent suitability index as areas of high (red) and low risk (blue). **(B)** Binary ecological niche model with reduced occurrences from filtering pseudoreplicated cases. This model was calibrated using principal components for a reduced number of variables. In this case, risk was defined as the presence of suitable conditions for rabies occurrence (red) with an omission error of 5%. From Hijmans et al. (2005), Bárcenas-Reyes et al. (2015), and Escobar (2016).

More points are always better during model calibration for more informed and less variable forecasts (Escobar et al., 2013), but a balance should exist between the number of geographic occurrences and the environmental representativeness of them. More important than the number of occurrences is their quality. Several studies have utilized all the available occurrence data of species in original format for model calibration without careful data curation (e.g., Brito-Hoyos et al., 2013; Bárcenas-Reyes et al., 2015). Indeed, failing to reduce pseudoreplicates (i.e., non-independent samples), and the consequent overrepresentation of environmental conditions, could produce models that simply reflect biases in the surveillance effort. **Figure 7** shows how a single environmental value could be overrepresented in a model due to sampling bias. In this example, it is evident how different data curation approaches and assumptions could vary in the use of occurrences from the original 45 occurrences to one occurrence per pixel or even a single pixel to summarize the same information. However, species found consistently in the same environmental space, with occurrences frequently falling in the same conditions, could be a classic case of an endemic specialist species of narrow niche (e.g., **Figure 6**). Thus, it is critical to differentiate between species with narrow niches and narrow niches resulting from sampling bias. For example, a forecast of bat-borne rabies in cattle in Mexico suffered model overfitting, resulting in the estimations of narrow areas with predictions of “high transmission risk” and areas with gaps of surveillance predicted of “low” risk (Bárcenas-Reyes et al., 2015). However, a reanalysis of the same data removing pseudoreplicate occurrences and redundant variables, showed broad areas that were now predicted to be at risk of rabies occurrence in cattle (**Figure 8**).

## Reports of Disease Absence

**FIGURE 9:**
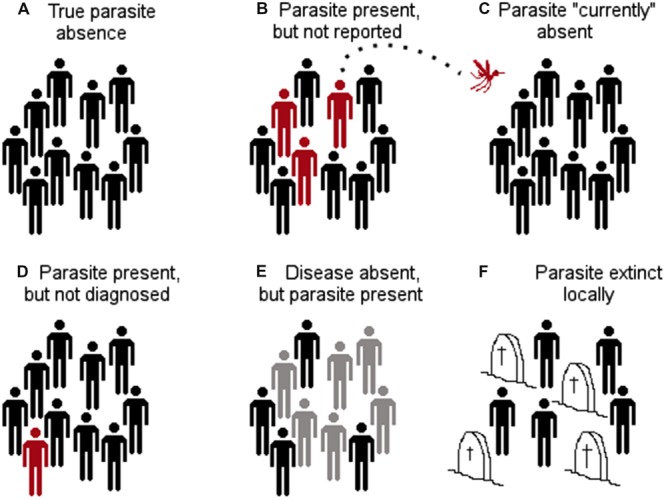
**Different configurations of parasite absence records. (A)** True parasite absence as a consequence of inhabitable conditions. E.g., absence of Tanapox virus in the Antarctic region due to the lack of suitable environmental conditions and absence of reservoirs and vectors (Monroe et al., 2014). **(B)** False absence: parasite present but not detected. E.g., in situations in which countries lack adequate epidemiological surveillance systems (e.g., Dominican Republic reports bat-borne rabies, while Haiti does not report this virus; both countries are located on the same island (Escobar et al., 2015a). **(C)** False absence: parasite currently absent, but environmental conditions are suitable and vectors and reservoirs are present. The parasite is likely to invade if translocated from an infected region to the free/suitable area (e.g., Chikungunya virus was absent in the Americas in early 2013, but was introduced later that year; Van Bortel et al., 2014). Thus, models considering the Americas as unsuitable due to the absence of the virus failed to consider the disease biogeography. **(D)** False absence: the parasite is present but not diagnosed due to low abundance or low sensitivity of diagnostic tests (e.g., Chikungunya virus may be present in some locations but due to the symptoms is referred as dengue infection). **(E)** False absence: the parasite is present in the host population, but there is no evidence of disease (e.g., host population has high immunity to avoid symptoms, however, shedding exists). **(F)** False absence: the parasite was or will be present, but at the time of the survey it was absent (e.g., suitable conditions for the parasite occurrence exist, but the parasite was extinguished due to control measures or the susceptible host population was removed by the parasite). Red: infected with the parasite. Gray: infected with the parasite but without symptoms. Black: not infected. Headstones: removed by the parasite.

Early models to link parasites with landscape features included logistic regressions. Logistic regressions, however, require the identification of locations with the presence and absence of the parasite. Most parasite presence data may be accurate in terms of taxonomic identification and georeferencing due to modern diagnostic methods and global positioning system devices; the correct identification of parasite’s absence may be, on the other hand, uncertain or incorrect (Peterson, 2014). Using incorrect absence data for model calibration may reduce the model fit by including locations where the parasite is or may be present but is reported as absent. A parasite may be reported as absent in a specific location due to many reasons (**Figure 9**). The parasite may be present in the host population, but it was simply undetected by the researcher (MacKenzie et al., 2002); the parasite may be present but it was eradicated recently; or the parasite could be present but biogeographic barriers do not allow it to use the suitable areas (**Figure 1**). Thus, calibrating ecological niche models of parasites, vectors, or hosts using absence data may fail to correctly capture the environmental signature of the target species.

Because absence data are hard to collect from the field, some approaches create dummy absence data sets in order to provide regression models with the absence data required for calibration. Some of these approaches include the random generation of virtual absences across the study area; such virtual absences are termed *pseudoabsences* and lack biological meaning (Lobo et al., 2007). To mitigate the error implicit in models requiring absence data, new algorithms are available for mapping diseases using only robust reports of parasites’ occurrence including presence-only and presence-background algorithms. Descriptions of such techniques have received broad attention and are broadly accepted by the scientific community (Franklin, 2009; Peterson et al., 2011; Pliscoff and Fuentes, 2011).

## Environmental Variables Used in Ecological Niche Modeling

**FIGURE 10:**
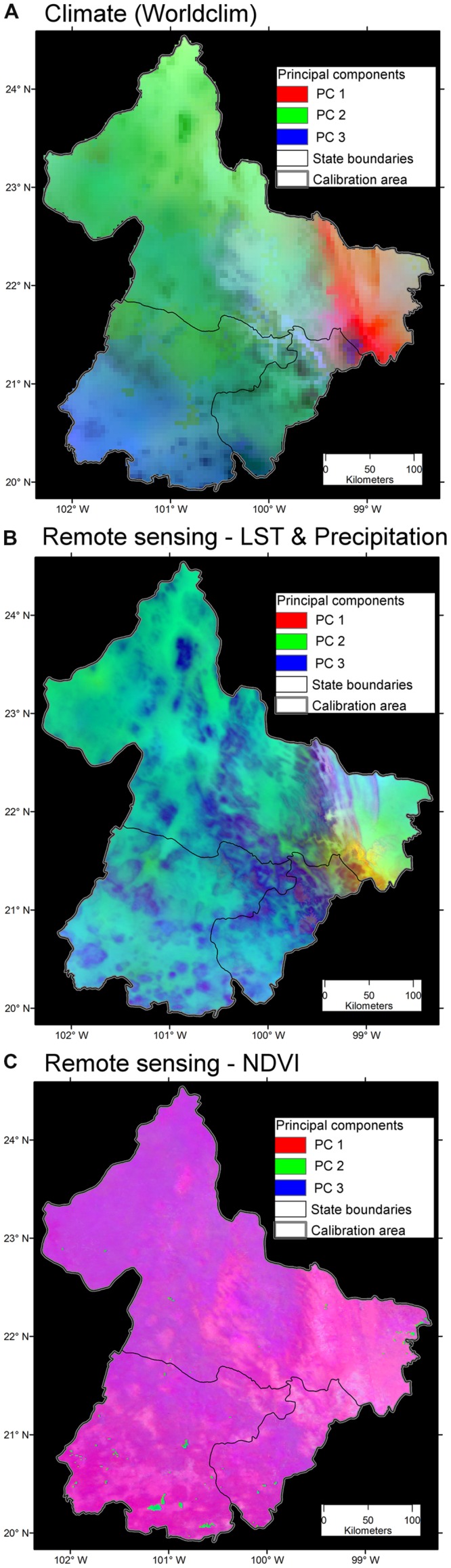
**Variability of three environmental data sets. (A)** Bioclimatic variables at ∼5 km resolution. Note broad areas with homogeneous environmental values (i.e., high spatial autocorrelation; continuous areas of similar color). The first three components accumulated 99.44% of the overall variance from the 19 variables. Data obtained from Hijmans et al. (2005). **(B)** Land surface temperature (LST) from satellite imagery and precipitation at ∼1 km. Note the increased heterogeneity of environmental values across the study area. The first three components accumulated 97.79% of the overall variance from 19 variables. Data obtained from http://worldgrids.org/ (Hengl et al., 2015). **(C)** Vegetation index, NDVI, from the AVHRR sensor at ∼1 km resolution. Note the high detail of information, even capturing areas with water bodies in the south (blue). The first three components accumulated 81.32% of the overall variance from 19 variables, thus, this data set provided less correlation between variables. Data obtained from UMD (2001). Principal components 1 (red), 2 (green) and 3 (blue) from the original variables. Source (Escobar, 2016).

**FIGURE 11:**
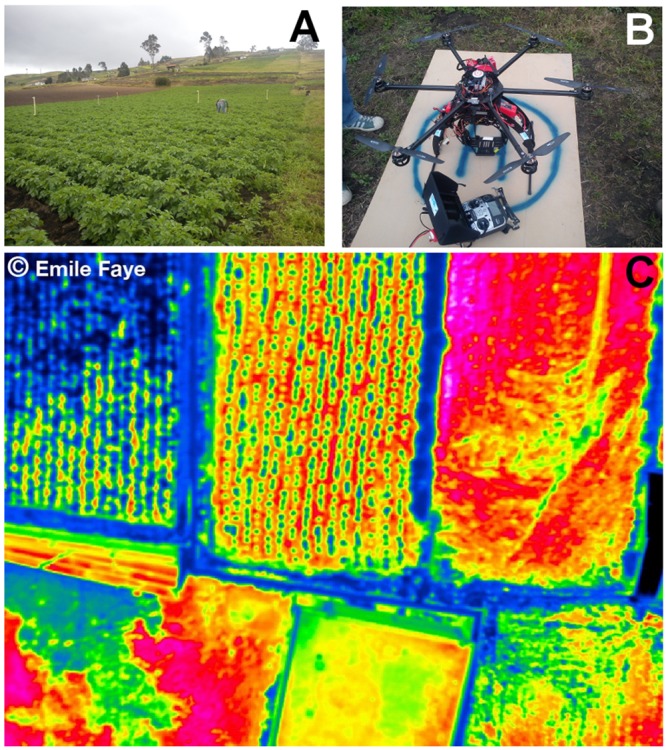
**Remote sensing data of temperature collected using drone technology. (A)** Potato crop field in Ecuador where thermal images were collected. **(B)** RC-drone PIXTIM, DJI Wookong-M autopilot with GPS receptor and 12 min flight autonomy used for data collection. **(C)** Resulting temperature image at 2 cm spatial resolution. Images courtesy: Danilo Yanez and Emile Faye. © Emile Faye – IRD.

Different environmental variables exist at diverse spatial and temporal resolutions (**Figure 10**). The environmental variables selected should respond to the specific scientific question and should consider the parasite’s biogeography, the spatial scale, the availability of parasite occurrence data, and the spatial and temporal match between occurrences and environmental variables. For global disease maps with considerable georeferencing error, environmental variables may include climate data, while for models at medium scale (i.e., continental-country size) with good referencing accuracy, remote sensing data could be a valuable source of environmental information to inform models (Peterson et al., 2011). At more fine scales (e.g., forest, host’s body) variables may not exist requiring their development by the modeler. Potential variables at a fine scale (i.e., a forest, cropland, town block) are typically not available, but drones capturing land reflectance are a promising tool to generate fine scale environmental grids with spatial resolutions at a centimeter scale (**Figure 11**). At a very fine scale (i.e., the host) environmental variables could include features of host’s skin, temperature range in the host surface, epithelia type, among others of crucial importance for the parasite to survive and maintain populations. However, biotic interactions (**B** from the **BAM** diagram; **Figure 1**) also should be considered at this microscale. Research on the distribution of parasites at the fine scale is still a neglected field and challenges include our limited understanding of competition, mutualism, and facilitation among parasites in the host and host immunity and behavior during novel or recurrent infections or co-infections.

**FIGURE 12:**
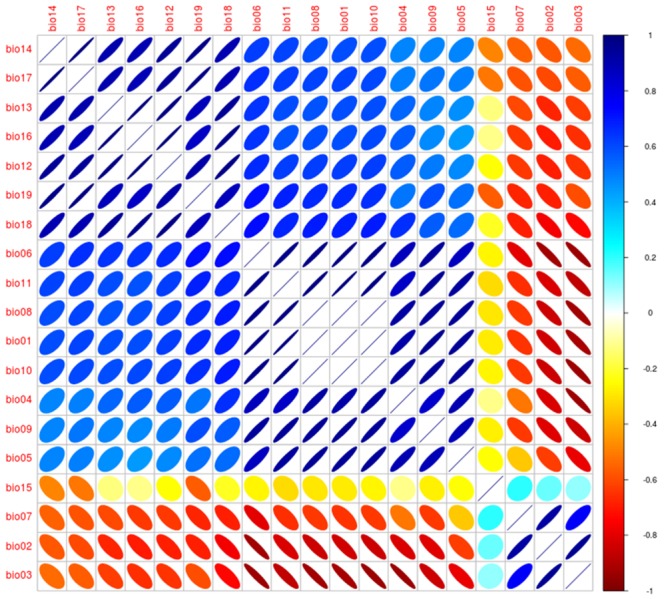
**Correlalogram from the Worldclim variables**. Correlation between variables was explored for the 19 Worldclim bioclimatic variables obtained from Hijmans et al. (2005) as in Escobar (2016). Size of ellipsoids denotes high (broad) and low (narrow) variability, direction of ellipsoids represents positive (right) or negative (left) association, colors denote correlation coefficient values to identify high negative (red), low (yellow), and high positive (blue). Analysis was developed using NicheToolBox (Osorio-Olvera, 2016).

The spatial scale must also be considered during variable selection. Climate data are often required at broad scales to capture environmental signatures of species across space. Disease maps generated at a local scale calibrated using climate data may fail to clearly identify patterns of parasite occurrence, as these data could be highly spatially autocorrelated at narrow extents (Peterson and Nakazawa, 2007). For example, a study of the spatial epidemiology of bat-borne rabies demonstrated that using climate as environmental space could be too coarse to explain the spatial distribution of vampire rabies in small countries (Escobar and Peterson, 2013). However, the *status quo* in ecological niche models in epidemiology is a default utilization of the 19 bioclimatic variables of Worldclim (Hijmans et al., 2005). In fact, Worldclim bioclimatic variables have high correlation (**Figure 12**), resulting in model overfitting and redundant information in the models (Peterson et al., 2011). The limits between spatial scales are fuzzy; more research in this arena is necessary given that most of the literature in ecological niches is based on one robust set of climatic variables with a decade of use (Hijmans et al., 2005). Thus, attention is needed to identify the spatial scale considered in each study (for more details in variable selection and data sources see Peterson et al., 2011; Peterson, 2014).

### Scale, Scale, Scale

Conceptual, methodological, or philosophical disputes may emerge when discussing the factors defining a parasite’s ecological niche. For instance, it is well known that in ecology debates arise when scientific questions are addressed at different spatial scales (Levin, 1992). In spatial epidemiology, debates dealing with different spatial scales also occur (Astorga et al., 2015b). For instance the environmental variables and assumptions required to describe a parasite’s ecological niche are highly dependent on the spatial scale.

The study of parasites may be expressed under diverse spatial scales. For example, at a micro scale rabies virus affects nervous tissues, in fact, rabies is diagnosed using samples from the brainstem and cerebellum in view of the high replication and detection of virus in these organs (Rupprecht et al., 2002). Thus, rabies virus is not distributed at random in the host, instead it occurs in specific tissues. Consequently, the environmental requirements of rabies may be tractable at this tiny scale. At a larger scale, rabies virus has a taxonomic signature, and even with a diversity of potential hosts, the virus can be perpetuated only in mammalian hosts, mainly Carnivora and Chiroptera. Thus, rabies is not distributed at random among all taxa and the physiological features of hosts have an environmental pattern for the virus that may be tractable (Gough and Jorgenson, 1976). Rabies virus can also have a landscape level signature. In bat-borne rabies, the environmental features required by the virus is a combination of soil, vegetation, and moisture requirements defining the ideal habitat to find an infected host (Escobar et al., 2013). Finally, at a more coarse scale, the potential distribution of rabies may be inferred at continental scales due to patterns of host occurrence under climate conditions (Kim et al., 2014).

Scale complexities also occur for other pathogens like *Pseudogymnoascus destructans* causing the white-nose syndrome in bats. The fungus affects the hairless skin of hibernacula bats of North America; again not distributed at random on the skin. This parasite has been found mainly in bats, and six bat species appear to be the most susceptible to the disease (Blehert et al., 2009). At a larger scale, conditions inside a cave may offer variations in humidity, temperature, wind speed, and substrate type that differ in the level of suitability for the fungus growth. At a different scale, the disease can also be tracked at landscape level including soil, climate, and landscape features associated with caves where the disease occurs (Flory et al., 2012). At a more continental scale, ecological niche modeling can be employed to understand patterns of distribution and invasion of this parasite based on climate conditions (Escobar et al., 2014).

Cholera for instance has a special affinity to the intestinal epithelia (Harris et al., 2012), thus, the environmental features *in vivo* can also be tracked. At another scale, pH, salinity, and temperature are associated with *V. cholerae* occurrence (Huq et al., 1984). At a coarser scale, *in situ* environmental requirements of this bacterium helped to predict its distribution in seawater environments at a global scale (Escobar et al., 2015c).

In a model of rabies in livestock in three states of Mexico, climatic data showed high homogeneity among neighbor cells as a result of the interpolative nature of these data (Escobar, 2016). In other words, climate data failed to capture fine scale patterns of environmental variability, suggesting that the study area was too small to calibrate an ecological niche model based on climate solely. When remote sensing (e.g., land surface temperature) and climate data (i.e., precipitation) were considered, the environmental conditions across the study area were more heterogeneous, capturing more information. However, at the same spatial resolution (1 km), remote sensing data summarizing primary productivity provided more landscape details (e.g., identification of water bodies; **Figure 10**). Thus, one should be aware of incorrect comparisons between models at different scales. In basic ecology, errors in conclusions associated with comparisons at different scales is termed “the Beale fallacy,” and has not been proposed until recently (Escobar et al., 2013; Peterson, 2014). One model calibrated at landscape scale may have a different pattern than a model developed at continental scale. Defining *a priori* the study area extent is also a crucial step during the study design of ecological niche models. Different study areas can generate different ecological niche model results (Barve et al., 2011). For example, Peterson and Samy (2016) recently proposed that a detailed selection of the study areas, to map Ebola in Africa, could be more informative, realistic, and robust than a model calibrated in the entire continent (Pigott et al., 2014b). Therefore, the study area extent should be strongly supported by ecological, instead of political, pragmatic, or administrative, reasons.

## Parasite, Vector, Reservoir: What to Model in the Disease System?

**FIGURE 13:**
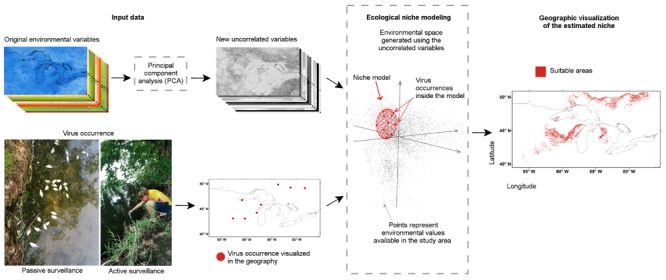
**Ecological niche modeling workflow for a “black-box” analysis**. Disease cases or reports of the pathogen from active or passive surveillance were used. **(Bottom left)** Information about the disease occurrence was generated based on passive or active surveillance, then (arrow), occurrences were curated to remove erroneous reports and obtain the final map of locations. **(Top left)** Environmental conditions of the study area were generated or collected from climate or remotely sensed data repositories, then (arrow), environmental variables were curated to remove redundant variables or erroneous layers. **(Center)** Environmental variables were used to create a multivariate environmental space where the niche of the species was estimated using the available occurrences of the parasite (dashed line box). **(Right)** Once the niche was defined, estimations were projected to the geographic space to construct suitability maps or geographic distributions models. Figure 1 from Escobar et al. (2016a) reproduced by permission of John Wiley and Sons.

Ecological niche modeling is a useful tool to understand the ecology of diseases caused by novel or poorly understood parasites (e.g., Ebola and Marburg viruses; Peterson et al., 2004). Researchers may need to identify the ecological factors driving epidemics (Bhatt et al., 2013), propose potential vector species in a disease system of unknown vectors (e.g., candidate vectors for Chagas disease in Brazil; Gurgel-Gonçalves et al., 2012), or to identify the best candidate species to be the reservoir of an emergent parasite (e.g., candidate reservoirs for Tanapox virus in equatorial Africa; Monroe et al., 2014). Thus, ecological niche models can be calibrated using parasites, vectors, or reservoir occurrences. We also could use reports of human or animal disease for modeling as they summarize the entire disease system (in ecological niche modeling termed black-box models *sensu* Peterson, 2007; **Figure 13**).

Once a parasite’s ecological niche has been characterized, this information can be used to anticipate suitable areas for the parasite outside the known range or in the future. This approach was described and patented by Peterson and Vieglais (2001), and today it is applied in spatial epidemiology to identify potential areas for epidemics (Peterson et al., 2004, 2014; Zhu and Peterson, 2014). Using a parasite’s niche to identify novel areas of potential spread is based on the assumption that its ecological niche will remain consistent through time. In simple terms, the ecological niche will not evolve. Empirical evidence supports the idea that ecological niche will remain consistent (Peterson et al., 1999; Peterson, 2006a, 2011; Warren et al., 2008). In fact, it is considered that a parasite’s niche remains constant even if strains change in virulence. For example, *Toxoplasma gondii* strains may increase in virulence after passage through animals; the niche in abiotic terms, however, remains (Pavlovsky, 1966). Abiotic changes in ecological niches at coarse scales are rare (Soberón and Peterson, 2011; Petitpierre et al., 2012). Ecological niche models usually suggest that diseases such as malaria (*Plasmodium* spp.; Peterson, 2009), leishmaniasis (Peterson and Shaw, 2003), and cholera (Escobar et al., 2015c) would increase their distribution under current climate change trends. How parasites adapt to novel environmental conditions and changes in virulence deserves future research, and experimental studies covering a long generational time of the parasite, more than “human” time, are necessary to understand niche evolution and changes in environmental tolerances of parasites. Such studies may be feasible in some taxa in view of their short generation time (e.g., bacteria).

## Risk Maps: What is Risk? How do we Map it?

**FIGURE 14:**
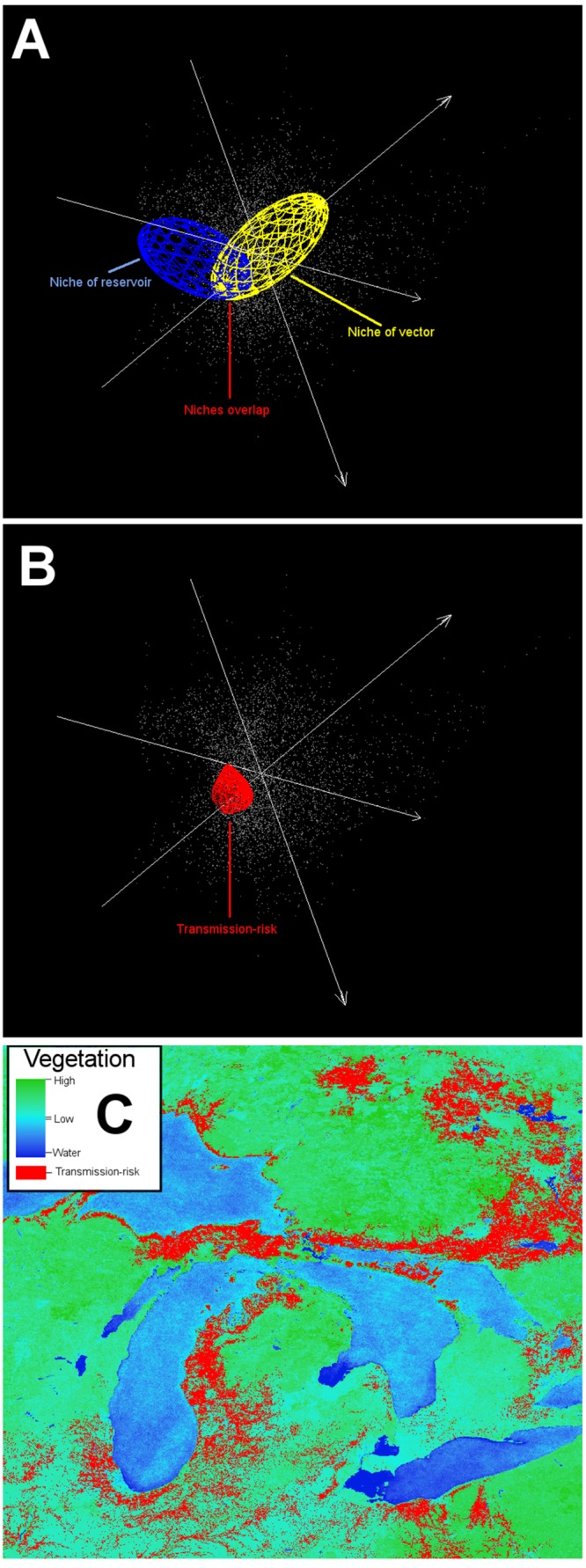
**Example of an ecological niche modeling application including a vector and a reservoir**. The ecological niche of a complex disease system may include a vector and a reservoir. Identifying the potential environmental overlap between the vector and the reservoir can inform potential areas for the effective pathogen occurrence. This scenario is more informative than the black-box approach, because more information is available (i.e., data about the vector and reservoir). **(A)** Ecological niche model of a vector (yellow ellipsoid) and a reservoir (blue ellipsoid). **(B)** Niche overlap between vector and reservoir (red polyhedron) denotes areas of potential pathogen cycle. **(C)** Projection of overlapping niches into the geographic space (red areas). Data obtained from http://worldgrids.org/ (Hengl et al., 2015).

Diseases could be a complex combination between the parasite’s abundance and strain, vector abundance and activity, and host immunity and force of infection. In fact, even when all the actors required in a disease system are present in a site, the disease may be absent, for example due to hosts with high immunity. Furthermore, defining the disease risk spatially is complex. Current literature, however, is crowded with the use of “risk” without a clear definition of the risk. Authors can overuse this concept in the title of manuscripts even when factors associated with risk are not contemplated in the study, making it difficult to identify literature related to mapping disease risk. When mapping disease risk, we suggest that risk should be quantifiable and defined for every study case, specifying if risk is proposed as: (i) the density of previous disease cases; (ii) the suitable areas for the occurrence of parasite, vector, or reservoir (**Figure 14**); or (iii) the factors associated with the susceptibility and vulnerability of the population of interest (e.g., low immunity, lack of health care, human behavior that facilitates transmission).

In ecological niche models, risk areas can be considered as areas with suitable environmental conditions for the occurrence of the parasite, vectors, and/or reservoirs (**Figure 14**). Risk delimitation in environmental terms can be complemented with factors acting at local scales. We strongly promote the use of the “disease-transmission risk” or “parasite-exposure risk” concepts, considering that even when the parasite is present in a population, disease *per se* may be absent (i.e., asymptomatic hosts) making the use of the term “disease risk” a strong assumption of exposure, infection, and symptomatology. Novel parasite discovery should not be considered as a true report of a pathogen (e.g., Bai et al., 2011), simply because recently discovered parasites may not be pathogenic. In the same context, a parasite found inside an arthropod does not prove that the arthropod acts as vector transmitting the parasite. But in both cases, risk may be “assumed” in terms of the potential of the parasite or the arthropod to participate in a disease system due to similarities (i.e., taxonomical, morphological, behavioral) with known pathogens or vectors. Noise also appears in reports of risk from emerging diseases. Emergent diseases could be hidden in the past but appear in modern times due to social circumstances, like an increment in surveillance effort, better diagnostic methods, or perhaps the entry of a new susceptible population to the parasite’s niche. Under this scenario, even when the risk of infection was always present in the population, there was no consideration of risk. In summary, the term risk must be defined in each study, as it is context dependent and because its assumptions and features change according to the population of interest. For public health, for example, risk could be generalized to: “No people, no risk.”

### Adding Risk Factors to Suitability Maps

Suitability maps of parasites could be complemented with information on factors associated with the facilitation of their transmission, including biotic interactions (**Figure 14**). Recently, Anderson (2016) discussed the positive influences that information resembling parasite/hosts interactions could have in ecological niche models, resulting in more detailed, place- and time-dependent, realistic predictions. For example, Samy et al. (2014) modeled the potential occurrence of Mycetoma disease, an infectious skin and bone disease that had been linked to the presence of trees from the *Acacia* genus. Mycetoma models were more accurate when *Acacia* records, a plausible tree reservoir, were added to the model. Also, Astorga et al. (2015a) used an ecological niche model of bat-borne rabies in Chile and, as a post processing step, added a dog-density surface to refine the predictive map and incorporate a risk dimension in terms of potential spillover of rabies from bats to dogs. The result was a risk map with an assertive and more informative forecast of bat rabies spillover events in dogs. Another example of supplementing an ecological niche model with variables of potential risk includes the use of air passenger flow between countries in view of the robustness of air traffic to explain epidemic spread (Brockmann and Helbing, 2013). Passenger flow via air transportation complemented a niche model for the identification of potential areas for chikungunya virus occurrence in countries across the Americas (Escobar et al., 2016b). Finally, a recent study used ecological niche modeling enriched with human density data and nighttime light satellite imagery to successfully estimate areas for human rabies transmission (Escobar et al., 2015b).

## Evaluation of Disease Maps

Spatial epidemiologists should acknowledge the effort of ecologists in developing tools, conceptual bases, and variables for ecological niche modeling. The body of literature for these methods has been, however, inspired mainly from the fields of ornithology and biological conservation (Franklin, 2009; Peterson et al., 2011). In this regard, disease mapping is different in limitations and assumptions because parasites occur in complex systems incorporating several species and because incorrect predictions may have negative implications in human and animal health. Models developed to guide public health interventions should have an intense and robust validation process before publication.

Contrasting with the large debate on algorithm performance and software development in ecological niche modeling for modeling biodiversity (Elith et al., 2006; Fitzpatrick et al., 2013; Blonder et al., 2014; Qiao et al., 2015b), little attention has been paid to the critical step of model evaluation (but see Muscarella et al., 2014). Nowadays, the gold standard test for ecological niche model performance uses the area under the curve (AUC) of the receiver operating characteristic (ROC) metric. AUC ROC *sensu stricto* employs parasite’s presence and absence for model evaluation. As described above, true absences are hard to obtain. To solve this problem, a common practice is to use virtual absences to feed the ROC metric, resulting in questionable evaluations (Lobo et al., 2007; Peterson et al., 2008; Golicher et al., 2012). This may be acceptable when modeling the potential distribution of, for example, an endangered plant or other non-lethal organism. But if the goal of an ecological niche model is to anticipate the potential distribution of Ebola or rabies viruses, models require deep assessment avoiding artificial data. Additionally, modelers use the AUC ROC metric to evaluate model predictions based on the points employed during model calibration, which may be not challenging for the algorithm, considering that points used to create the model are used to validate it, thereby lacking statistical independence (Hurlbert, 1984). The AUC ROC metric also fails to identify those models that over predict the potential areas for disease occurrence (which is not too bad) or those models that under estimate the areas in which suitable conditions for the parasite exist, but the model simply neglects them (which is dangerous for virulent parasites) (see Lobo et al., 2007).

In a recent study, researchers used ecological niche modeling and a detailed set of *Aedes aegypti* and *A. albopictus* occurrences to determine the spatial limits of dengue fever and chikungunya at global scale (Kraemer et al., 2015). The model resulted in important inferences about the potential distribution of these vectors. The model evaluation process was based on a metric requiring presence and absence data, but absence data were not available. To solve this, authors generated their own absence data via random points across the world, reducing the robustness of the test. The study generated models that mainly reflect the areas with reports (i.e., high model overfit). In this study, Chile, for example, was predicted unsuitable for *A. aegypti*, but the current lack of vector reports in this country is the result of aggressive efforts of authorities for vector eradication and active epidemiological surveillance. *A. aegypti*, however, has been recorded recently, again, in Chile in Arica and Camarones with reports including adult female mosquitoes and larvae (Instituto de Salud Publica, 2016).

Three alternative metrics can be employed to evaluate presence-only ecological niche models including Akaike information criterion (Warren and Seifert, 2011), cumulative binomial probability test (CBP; i.e., identifies if models are predicting occurrences better than by random using an independent set of occurrence data not employed during model calibration) (Peterson et al., 2011), and Partial ROC, a new metric incorporating both ROC AUC and CBP approaches (Peterson et al., 2008; Peterson, 2012; Escobar et al., 2013). Nevertheless, more efforts are needed to test the abilities of these and new metrics to discriminate among different model hypotheses.

## Final Remarks

In this manuscript we describe how ecology, especially the parasite’s ecological niche, is key to understanding the biogeography of disease systems. Ecological niche models of parasites may help us to respond to ecological and distributional questions related to epidemic potential. However, epidemiology has largely failed to adopt the conceptual bases that help to correctly design and interpret ecological niche models for disease mapping.

The use of ecological niche modeling methods for disease mapping should be based on a clear understanding of the **BAM** framework and its diversity of plausible configurations (Peterson, 2008; Saupe et al., 2012). Strikingly, click-and-run software dominates the ecological niche modeling practice and users argue that their selection of the method was “because [it] had been validated in peer-review publications,” showing that modelers basically develop predictions without a clear understanding of the process (Joppa et al., 2013). In epidemiology, several ecological niche models have been generated through a “recipe” without a clear justification of the study area extent and inclusion criteria for the data employed (e.g., Abedi-Astaneh et al., 2015; Ali Hanafi-Bojd et al., 2015; Hanafi-Bojd et al., 2015; Gholamrezaei et al., 2016). This practice has been criticized (Anderson, 2014) and more detailed study designs have been encouraged (Peterson, 2014).

Five main questions have been identified for the study design of ecological niche modeling of diseases (Escobar, 2016): (i) Which occurrences to use and why? (e.g., pathogen or reservoir, occurrence inclusion criteria) (ii) Where to calibrate the models and why? (i.e., study area extent) (iii) Which variables should be employed and why? (iv) What algorithms will be explored and why? and (v) How models will be evaluated and why? (e.g., evaluations based on information theory or independent data sets). These questions could help to guide early stages of study designs and could be a helpful tool for readers and reviewers aiming to differentiate between good and incomplete research. Answers to these questions must be based on the research question, the empirical data available, and the natural history of the disease. Here we have explained how the ecological niche of parasites could be studied at different spatial scales, but parameters and variables required need to be generated at very fine scales. The results of the models could be used to map areas of potential transmission risk. Risk is a complex term, but to facilitate its utilization in spatial epidemiology, it should be defined and quantified clearly in each study case. Control and eradication of diseases demand first an understanding of its niche to interrupt the system on any stage or component. Ecological niche modeling shows a promising future in modern epidemiology, but their usefulness lays on the quantitative robustness and biological realism of their products.

## Author Contributions

LE conceived and designed the idea of the manuscript, performed the analyses, and wrote the paper; MC co-wrote the paper.

## Conflict of Interest Statement

The authors declare that the research was conducted in the absence of any commercial or financial relationships that could be construed as a potential conflict of interest.

## Footnotes

1 www.healthmap.org
